# Histological processing of un-/cellularized thermosensitive electrospun scaffolds

**DOI:** 10.1007/s00418-018-1757-7

**Published:** 2018-12-17

**Authors:** Julia Fuchs, Marc Mueller, Christine Daxböck, Manuela Stückler, Ingrid Lang, Gerd Leitinger, Elisabeth Bock, Amin El-Heliebi, Gerit Moser, Birgit Glasmacher, Dagmar Brislinger

**Affiliations:** 1 Department of Cell Biology, Histology and Embryology, Gottfried Schatz Research Center, Medical University of Graz, Neue Stiftingtalstraße 6, 8010 Graz, Austria; 2 Institute for Multiphase Processes, Leibniz University Hannover, Callinstraße 36, 30167 Hannover, Germany

**Keywords:** PCL, Cellularized prosthesis, Polycaprolactone, Polylactide acid, Graft, Paraffin embedding

## Abstract

Histological processing of thermosensitive electrospun poly(ε-caprolactone)/poly(l-lactide) (PCL/PLA) scaffolds fails, as poly(ε-caprolactone) (PCL) is characterized by its low-melting temperature (Tm = 60 °C). Here, we present an optimized low-temperature preparation method for the histological processing of un-/cellularized thermosensitive PCL/PLA scaffolds.

Our study is aimed at the establishment of an optimized dehydration and low-melting-point paraffin-embedding method of electrospun PCL/PLA scaffolds (un-/cellularized). Furthermore, we compared this method with (a) automatized dehydration and standard paraffin embedding, (b) gelatin embedding followed by automatized dehydration and standard paraffin embedding, (c) cryofixation, and (d) acrylic resin embedding methods. We investigated pepsin and proteinase K antigen retrieval for their efficiency in epitope demasking at low temperatures and evaluated protocols for immunohistochemistry and immunofluorescence for cytokeratin 7 (CK7) and in situ padlock probe technology for beta actin (ACTB). Optimized dehydration and low-melting-point paraffin embedding preserved the PCL/PLA scaffold, as the diameter and structure of its fibers were unchanged. Cells attached to the PCL/PLA scaffolds showed limited alterations in size and morphology compared to control. Epitope demasking by enzymatic pepsin digestion and immunostaining of CK7 displayed an invasion of attached cells into the scaffold. Expression of ACTB and CK7 was shown by a combination of mRNA-based in situ padlock probe technology and immunofluorescence. In contrast, gelatin stabilization followed by standard paraffin embedding led to an overall shrinkage and melting of fibers, and therefore, no further analysis was possible. Acrylic resin embedding and cyrofixation caused fiber structures that were nearly unchanged in size and diameter. However, acrylic resin-embedded scaffolds are limited to 3 µm sections, whereas cyrofixation led to a reduction of the cell size by 14% compared to low-melting paraffin embedding. The combination of low-melting-point paraffin embedding and pepsin digestion as an antigen retrieval method offers a successful opportunity for histological investigations in thermosensitive specimens.

## Introduction

Poly(ε-caprolactone)/poly(l-lactide) (PCL/PLA) scaffolds are a focus of tissue engineering as a promising substitute for expanded polytetrafluoroethylene (ePTFE) scaffolds (Siddiqui et al. 2018; Pfeiffer et al. 2014). The biodegradable scaffolds are used to support cell growth, thus recreating a functional microenvironment that stimulates organization of various tissues (Li et al. 2017; Shahrezaee et al. 2018). PCL/PLA scaffolds offer a wide range of possibilities due to porosity and surface modifications. Nevertheless, PCL is thermosensitive and has a melting point (Tm = 60 °C) much below the temperatures reached in conventional paraffin embedding and histological processing. The high melting point of PLA (Tm = 180 °C) in combination with PCL does not increase the feasibility of the material for histological processing and automatized dehydration, and standard paraffin embedding of PCL/PLA scaffolds has resulted in the dissolution of thermosensitive material (Azimi et al. 2014).

PCL/PLA scaffolds act as a three-dimensional (3D) microenvironment for cell adhesion, proliferation, differentiation, and extracellular matrix (ECM) formation and, therefore, mimic the conditions that allow recreating the native tissue (Dhandayuthapani et al. 2011). Various types of PCL/PLA scaffolds and applications are under investigation; e.g., electrospun PCL/PLA blend nanofibrous scaffolds to facilitate new bone formation, small-diameter fibrous vascular grafts, and nerve tissue regeneration (Yao et al. 2017; Prabhakaran et al. 2008). Immunocytochemistry in combination with laser scanning electron microscopy and scanning electron microscopy (SEM) is usually the methods of choice to prove that there are cellular adhesion, proliferation, and differentiation on the PCL/PLA scaffolds. Histological examinations of in vivo systems using implanted PCL/PLA scaffolds showed that cells intruded into the PCL/PLA scaffolds seem to increase the thermostability of the material (Shao et al. 2006; Gredes et al. 2017). Nevertheless, there is an obvious lack of appropriate histological processing methods for thermosensitive materials such as PCL/PLA scaffolds used for in vitro experiments.

There have been several attempts to overcome these difficulties in the histological processing of thermosensitive materials, e.g., by cryofixation and cryosectioning (Espandar et al. 2012), encapsulation in thermoreversible gels (Yasuda et al. 2006) or gelatin embedding prior to histological preparation (Hruschka et al. 2013).

The objective of this study was to establish a method for histological processing of un-/cellularized thermosensitive electrospun PCL/PLA scaffolds and to compare our method with already available histological processing procedures.

## Materials and methods

### Multilayer graft fabrication

Vascular scaffolds were fabricated using PCL (Mn = 70,000–90,000, Sigma-Aldrich, Taufenkirchen, Germany) and PLA (poly-l-lactic acid, Mw = 150,000, Natureplast, Ifs, France) polymers. The inner layer was spun from a blend of PCL (100 mg/ml) and PLA (50 mg/ml), while the outer layer was spun from pure PCL (200 mg/ml). Polymers were dissolved in 2,2,2-trifluoroethanol (Sigma-Aldrich, Taufenkirchen, Germany) with a stirrer for at least 24 h.

The electrospinning process was performed with a custom-made setup that consisted of a high-voltage power supply, a polymer reservoir with a 0.8 mm hollow needle as a nozzle, and a grounded collector. A mandrel of stainless steel with a diameter of 4 mm, rotating at 1000 rpm, was used as a collector. The applied voltage was set to 20 kV, and the distance between the nozzle and collector was 28.5 cm. Each layer was spun for 5 min at a flow rate of 3 ml/h.

### Material characterization

**Fig. 1:**
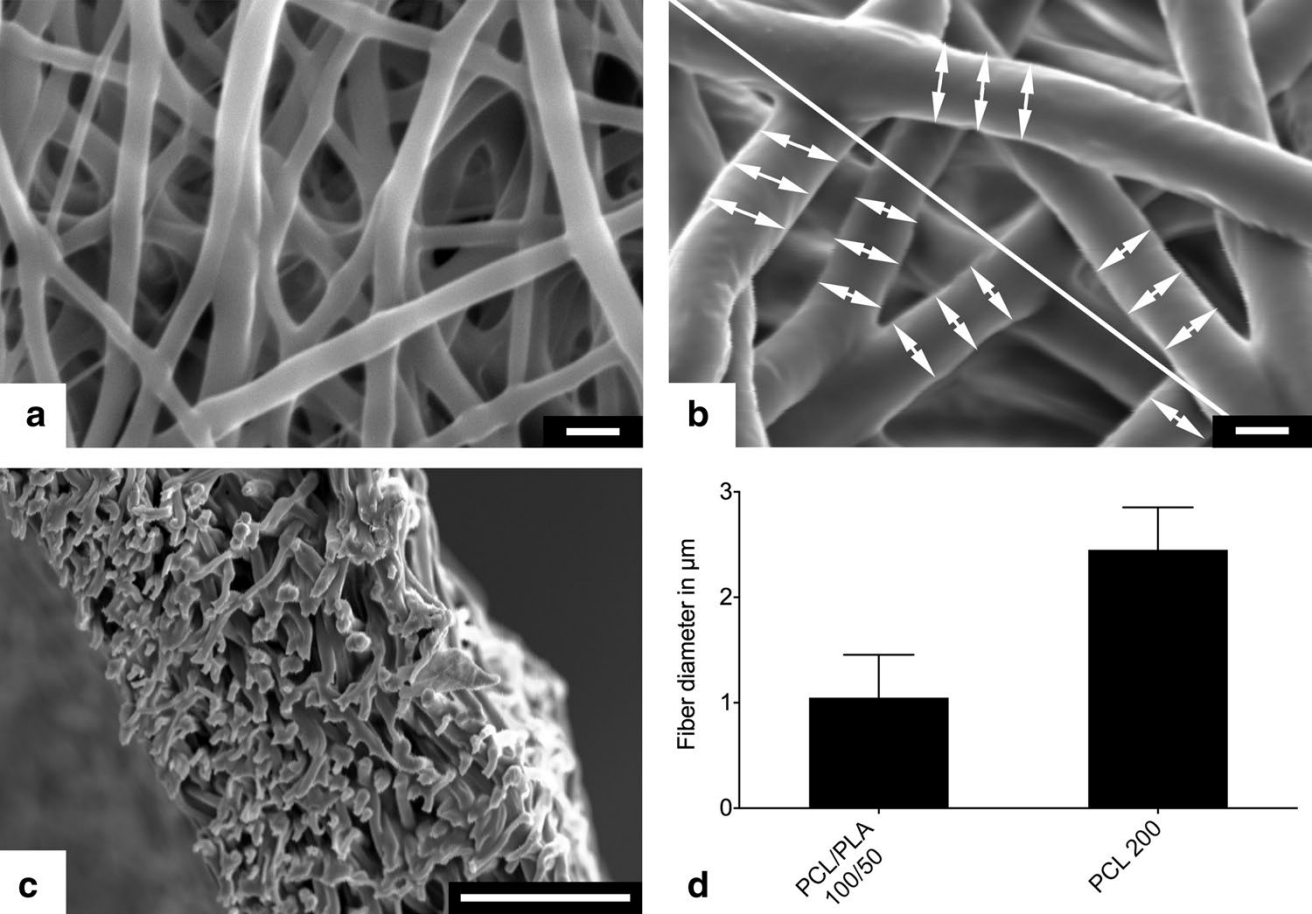
SEM images of the inner PCL/PLA layer (**a**) and outer PCL layer (**b**). All fibers crossing a diagonal line were measured on three spots left and right of the crossing (**b**). Wall cross sections showed no delamination of both layers (**c**). Fibers of the densely packed inner layer showed a mean diameter of 1.05 µm, whereas fibers of the highly porous outer layer had diameter of 2.45 µm (*n* = 1600)

The fiber diameters of the inner and outer layer grafts were measured using scanning electron microscopy (VP-SEM S3400, Hitachi Europe, Krefeld, Germany). Samples were obtained from eight different scaffolds and coated with gold–palladium using a sputter coater (Sputter Coater SC7620, Fa. Emetich) for 45 s at a distance of 5 cm. Five images per sample were acquired, resulting in a total number of 40 images for each layer of the scaffold. Images were acquired at an accelerating voltage of 15 kV, a distance of 7 mm (eucentric), and a magnification of 4000-fold. A line was drawn from the top left to the bottom right of the SEM image. Fiber diameters were measured, where the line crossed the fiber using AxioVision 4.7 (Carl Zeiss, Jena, Germany) with three measurements left and right from the diagonal line, respectively (Fig. 1). At least ten fibers were measured per image.

### Cell culture

Human first trimester trophoblast cell line ACH-3P (Hiden et al. 2007) was kindly provided by Gernot Desoye (Department of Obstetrics and Gynecology, Medical University Graz, Austria). Cells were cultured in low-glucose Dulbecco’s modified Eagle medium: Nutrient Mixture F-12 (Sigma-Aldrich, St. Louis, MO, USA) supplemented with 10% fetal bovine serum (FBS; Thermo Fisher Scientific, Waltham, Massachusetts, USA), 1% l-glutamine (200 mM; Thermo Fisher Scientific, Waltham, Massachusetts, USA), and 1% penicillin–streptomycin (PS; Thermo Fisher Scientific, Waltham, Massachusetts, USA). All cells were cultivated in a 95% humidified atmosphere at 37 °C and 5% CO_2_. Cells were counted using the automated CASY^®^ Cell Counter + Analyzer (Innovatis AG, Bielefeld, Germany).

### Preparation of uncellularized and cellularized PCL/PLA scaffolds

The PCL/PLA scaffolds were sterilized by gamma irradiation with doses of 25 kGy in accordance with the EN 13,485 and ISO11137 criteria (MEDISCAN, Kremsmünster, Austria). Sterile tubular PCL/PLA scaffolds with a length of 50 mm and an inner diameter of 4 mm were used and defined as uncellularized. For the preparation of cellularized PCL/PLA scaffolds, sterilized tubular PCL/PLA scaffolds were fixed in a polycarbonate housing with luer lock connections. Scaffolds were coated with 20 µg/ml fibronectin (Sigma-Aldrich, St. Louis, MO, USA) from human foreskin fibroblasts that were diluted in PBS to a final volume of 10 ml and were air-dried for 1 h at 37 °C before the cells were seeded. The outer scaffold surface was seeded with ACH-3P cells by filling the polycarbonate housing with a 15 ml cell suspension (5 × 10^5^ cells/ml). Nonattached cells were removed after being allowed to attach for 5 h at 37 °C. To achieve a homogenous cell colonization on the outer scaffold surface, a second seeding step was performed. For this, the graft was rotated 180°, and the ACH-3P cell suspension (5 × 10^5^ cells/ml) was filled into the polycarbonate housing. Nonattached cells were removed after 5 h at 37 °C. The attached ACH-3P cells were cultured in growth medium for 14 days without a medium change.

### Cryofixation and cryosectioning of un-/cellularized PCL/PLA scaffolds

Un-/cellularized PCL/PLA scaffolds were fixed in 3.7% paraformaldehyde (PFA; Diapath S.P.A., Martinengo BG, Italy) for 15 min and then were washed in 1 × PBS. Cryosections were produced by embedding the membranes in Tissue-Tek^®^ O.C.T. Compound at − 20 °C (Sakura^®^, Alphen aan den Rijn, The Netherlands) that were then sectioned on a cryotome (Microm HM 560; Histocom, Zug, Switzerland) to slices of 7 µm and mounted on SuperFrost Plus™ slides (Thermo Fisher Scientific, Waltham, Massachusetts, USA). Sections were air-dried for 24 h and stored at − 20 °C until they were stained. Hematoxylin and eosin (HE) staining was performed on the cellularized scaffolds.

### Automatized dehydration and standard paraffin embedding of un-/cellularized PCL/PLA scaffolds

Un-/cellularized PCL/PLA scaffolds were fixed in 3.7% PFA for 15 min and washed in 1 × PBS. Automatized dehydration and tissue processing for paraffin embedding of fixed PCL/PLA scaffolds was performed with a Tissue-Tek^®^ VIP^®^ 5 Vacuum Infiltration Processor (Sakura^®^, Alphen aan den Rijn, The Netherlands) according to conditions, as listed in Table 1. Standard paraffin embedding of the scaffolds was performed with standard paraffin wax (melting point at a minimum 56 °C; Vogel GmbH & Co. KG, Fernwald, Germany) with the Dispenser Unit of the paraffin-embedding center TES Valida^®^ (MEDITE Cancer Diagnostics, Orlando, USA). The paraffin in the paraffin tank was heated to 61 °C to avoid rapid cooling of the standard paraffin while embedding.

**Table 1:** Protocol for tissue dehydration and standard paraffin embedding used with the Tissue-Tek^®^ VIP^®^ 5 vacuum infiltration processor

	Time (min)	Temperature (°C)
Paraformaldehyde (3.7%)	0	40
60% ethanol	60	40
80% ethanol	60	40
96% ethanol	60	40
100% ethanol	60	40
100% ethanol	60	40
100% ethanol	60	40
Tissue clear	60	40
Tissue clear	60	40
Tissue clear	60	40
Paraffin (56 °C)	60	56
Paraffin (56 °C)	60	56
Paraffin (56 °C)	60	56
Embedding		61

### Gelatin embedding (5%, 10%, and 25%) followed by automatized dehydration and standard paraffin embedding of un-/cellularized PCL/PLA scaffolds

Un-/cellularized scaffolds were fixed in 3.7% PFA for 15 min and washed with 1 × PBS. Samples were incubated in 5% gelatin (bovine skin; Sigma-Aldrich, St. Louis, MO, USA) for 30 min at 37 °C followed by an incubation step in 5%, 10%, or 25% gelatin solution for 30 min at 37 °C. Afterwards, the samples were incubated for 15 min at 4 °C to harden the gelatin-scaffold block. The construct was transferred to a larger well, prewarmed gelatin solution (5%, 10%, or 25%) was added, and the samples were cooled for 15 min at 4 °C. The blocks were transferred into a 3.7% PFA solution [room temperature (RT), 24 h] and washed in 1 × PBS at RT. Finally, the PFA-fixed and gelatin-stabilized PCL/PLA scaffolds were embedded in standard paraffin with a Tissue-Tek^®^ VIP^®^ 5 Vacuum Infiltration Processor (Sakura^®^, Alphen aan den Rijn, The Netherlands) and TES Valida (MEDITE Cancer Diagnostics, Orlando, USA) using the parameters, as listed in Table 1.

Formalin-fixed-paraffin-embedded (FFPE) blocks were cut into 7 µm sections using an automated rotary microtome (HM355 with STS & Cool-Cut, Thermo Fisher Scientific, Waltham, Massachusetts, USA), and the sections were then transferred to SuperFrost Plus™ slides and dried overnight at 45 °C. HE staining was performed on cellularized scaffolds.

### Low-melting-point paraffin embedding of un-/cellularized PCL/PLA scaffolds

Un-/cellularized scaffolds were fixed in 3.7% PFA for 15 min and washed with 1 × PBS. Dehydration and low-melting-point paraffin (melting point 50 °C; Carl Roth, Karlsruhe, Germany) embedding of fixed PCL/PLA scaffolds was performed with a KOS Microwave Multifunctional Tissue Processor (Milestone, Sorisole, Italy). Embedding conditions are listed in Table 2. Embedded scaffolds were cut to sections of 7 µm and transferred to SuperFrost Plus™ slides and dried overnight at 45 °C. HE staining was performed on cellularized scaffolds.

**Table 2:** Protocol for tissue dehydration and low-melting-point paraffin embedding used with the KOS microwave multifunctional tissue processor

	Time (min)	Temperature (°C)
100% ethanol	13	45
Isopropanol	13	45
Low-melting paraffin (50 °C)	23	50
Embedding		

### Acrylic resin embedding of un-/cellularized PCL/PLA scaffolds

Un-/cellularized PCL/PLA scaffolds were fixed in 3.7% PFA for 15 min and washed with 1 × PBS. Specimens were dehydrated with an ethanol series of 50%, 70–96% for 15 min each. The scaffold was then treated with 96% ethanol and acrylic resin (LR white; 1:1; Sigma-Aldrich, St. Louis, MO, USA) for 1 h, followed by two incubations with LR white resin for 1 h. The scaffolds were manually embedded in LR white resin in airtight gelatin capsules (Pohl-Boskamp GmbH & Co. KG, Hohenlockstedt, Germany). For polymerization, the closed capsules were incubated for 3 days at 45 °C. LR white resin blocks were cut into 3 µm sections using a Leica EM UC7 ultramicrotome (Leica Biosystems, Wetzlar, Germany) and mounted on SuperFrost Plus™ slides for 3 h at 47 °C. Cellularized scaffolds were analyzed with toluidine blue staining (Agar Scientific, Essex, UK).

### Sample preparation and enzymatic antigen retrieval for immunological analyses

Low-melting-point paraffin sections (7 µm) mounted on SuperFrost Plus™ slides were dewaxed using Histolab Clear^®^ (Histolab^®^, Askim, Sweden) solution four times for 5 min each and then rehydrated in a graded series of 100%, 96%, and 70–50% ethanol, followed by three washing steps in distilled water for 3 min each. An enzymatic antigen retrieval with pepsin was performed by incubating cellularized membrane sections with 2 mg/ml pepsin (Sigma-Aldrich, St. Louis, MO, USA) in 0.1 M HCl (Sigma-Aldrich, St. Louis, MO, USA) for 30 min at 37 °C. Enzymatic antigen retrieval with proteinase K was performed by incubating cellularized membrane sections with 20 µg/ml proteinase K (Roche, Basel, Switzerland) in TE buffer (pH 8.0) (Ramos-Vara and Beissenherz 2000). Both treatments were performed in a humidified chamber. The digestions were stopped by washing the slides with distilled water for 2 min. For application of the in situ detection padlock probe method, samples were dehydrated using an ethanol series of 70%, 85%, and 99.5% for 1 min each and stored at − 80 °C until use.

### Immunohistochemistry

Immunostaining was performed using an UltraVision Detection System HRP Polymer Kit (Thermo Fisher Scientific, Waltham, Massachusetts, USA) according to the manufacturer’s protocol. In brief, endogenous peroxidase was blocked using a hydrogen peroxidase block for 10 min. After three washing steps with 1 × TBS, background blocking was performed using the Ultra Vision Protein Block for 5 min. Polyclonal rabbit Cytokeratin 7 (CK7)—antibody (AP06204PU-N; Acris, Herford, Germany) was diluted 1:200 to a final concentration of 5 µg/ml in antibody diluent (Agilent Technologies, Santa Clara, CA, USA) and incubated on slides for 30 min at RT. Slides were then washed three times, and detection was achieved by incubation with an anti-rabbit UltraVision HRP-labeled polymer for 15 min and 3-amino-9-ethylcarbazole (AEC; Thermo Fisher Scientific, Waltham, Massachusetts, USA) for 10 min. Nuclei were stained with hematoxylin, and slides were aqueous mounted with Kaiser’s glycerol gelatin (Merk Millipore, Darmstadt, Germany). Rabbit IgG (X0936; 1:200; 5 µg/ml; Agilent Technologies, Santa Clara, CA, USA) served as a negative control.

### Immunofluorescence

Immunofluorescence was performed using a goat–anti-rabbit antibody conjugated with Alexa Flour 633 (A21070; Thermo Fisher Scientific, Waltham, Massachusetts, USA) for detection of CK7 for the visualization of ACH-3P cells.

Sections with seeded ACH-3P were washed with 1 × PBS and incubated with UV Block (Thermo Fisher Scientific, Waltham, Massachusetts, USA) for 10 min. The primary antibody, polyclonal rabbit CK7, was diluted 1:200 in antibody diluent and incubated for 30 min. Subsequently, slides were washed with 1 × PBS and incubated with the secondary antibody, Alexa Fluor 633 goat–anti-rabbit (1:200; Thermo Fisher Scientific, Waltham, Massachusetts, USA) for 30 min. Finally, the slides were washed, and the nuclei were stained with DAPI (1:2000; Thermo Fisher Scientific, Waltham, Massachusetts, USA) for 5 min. Rabbit immunoglobulin fraction (5 µg/ml, diluted in antibody diluent, Agilent Technologies) served as a negative control. Sections were mounted with ProLong™ Gold Antifade Reagent (Thermo Fisher Scientific, Waltham, Massachusetts, USA).

### In situ padlock probe technology and immunofluorescence

Oligonucleotides for beta actin were used as previously published (Grundberg et al. 2013; El-Heliebi et al. 2017). The ACTB padlock probe was ordered to be 5′-phosphorylated (Integrated DNA Technologies, Coralville, IA, USA). The LNA primer was purchased from Exiqon (Exiqon, Vedbaek, Denmark) and the detection probe from Biomers (Biomers, Ulm, Germany). The LNA-primer-, padlock probe-, and detection probe sequences are listed in Table 3.

**Table 3:** Oligonucleotide sequences for in situ padlock probe technology

Primer	Sequence (5′–3′)
ACTB*	C+GG+GC+GG+CG+GATCGGCAAAG

Padlock probe	Sequence (5′–3′)
plp_ACTB*	AGCCTCGCCTTTGCCTTCCTTTTACGA**CCTCAATGCACATGTTTGGCTCC**TCTTCGCCCCGCGAGCACAG

Detection probe	Sequence (5′–3′)
D1_ATTO 550	ATTO 448-**CCTCAATGCACATGTTTGGCTCC**

Padlock probe was 5′-phosphorylated. The fluorophore of the detection oligonucleotide was 5′ conjugated

+: the following base is LNA modified; underlined: target complement sequence, bold: the complementary detection probe sequence

Antigen retrieval of the cellularized membranes was performed by pepsin digestion as previously described. All in situ reactions were performed in secure-seals hybridization chambers (Sigma-Aldrich, St. Louis, MO, USA) with a volume of 50 µl according to the method described by El-Heliebi et al. (El-Heliebi et al. 2017). In addition to single staining, padlock probes for targeting the housekeeping gene ACTB were combined with immunofluorescence staining for CK7. Therefore, immunofluorescence for CK7 was performed immediately after the in situ padlock probe ligation step as described elsewhere (El-Heliebi et al. 2018). To continue the in situ padlock probe method, the rolling circle amplification, as well as detection probe hybridization step, was applied after IF staining. Reverse transcription master mix without TranscriptMe reverse transcriptase (DNA-Gdansk, Poland) served as a negative control.

### Image acquisition and analysis

We used the visibility of fiber structure and discrimination of coarse- and fine-meshed structures as parameters to assess the qualitative outcome of the processed scaffolds for the comparison of the selected methods.

Analysis was performed by scanning the cellularized scaffolds to take 20 × and 40 × microscope images. Brightfield and fluorescence images were acquired using the Zeiss Observer.Z1 inverted microscope (Carl Zeiss, Oberkochen, Germany) equipped with a 120 W HXP Mercury short-arc lamp. For brightfield images, the Axiocam 506 camera (Carl Zeiss, Oberkochen, Germany) was used, and fluorescence images were taken with the Axiocam 702 mono (Carl Zeiss, Oberkochen, Germany) equipped with an excitation and emission filter set for visualization of DAPI, FITC, Cy3, and Cy5. The 40 × objective (LD Achroplan 40 ×/0.60 corr., *D* = 0–2 mm; Carl Zeiss, Oberkochen, Germany) and the 20 × objective (LD Plan-Neofluar 20×/0.40 corr., *D* = 0–1.5 mm; Carl Zeiss, Oberkochen, Germany), and the ZEN 2 blue software (Version 2.0.0.04.8.2.0; Carl Zeiss, Oberkochen, Germany) was used for capturing both the brightfield and fluorescence images. For enhanced visualization, brightness and contrast of each image was adjusted. Overlay images of fluorescence and brightfield channels were generated using the open-source GNU Image Manipulation Program (GIMP) version 2.8.22 (open-source software, http://www.gimp.org).

We used the open-source cell image analysis software CellProfiler (version 3.1.5, Carpenter et al. 2006) to evaluate the cell size of the attached cells on the processed cellularized PCL/PLA scaffolds. The pipeline was designed for quantification of the cell size by staining the intermediate filaments by CK7. Briefly, DAPI images were processed with the “Identify Primary Objects” module to identify the number of cell nuclei. The modules “Identify Secondary Object” and “Measure Image Area Occupied” were used for the measurement of CK7, displaying the cell area. Unspecific signals, which were simultaneously detectable in at least two fluorescent channels, were filtered by the “MaskObject” module. Overlay images of the evaluated cell area and nuclei outlines (Fig. 7b, d) were created using the modules “Overlay Objects” on Cy5 channel images combined with the module “Overlay Outlines” of DAPI objects. We have taken images in the Cy5 channel and used their pixel values to calculate the cell size [pixel/area ratio = 0.054289 µm^2^ for images of low-melting-point embedded sections (40 ×) and pixel/area ratio = 0.085849 µm^2^ for images of cryofixation embedded sections (20 ×)] in relation to the number of nuclei (Fig. 7).

### Statistical analyses

The experiments were performed 2 to 5 times to exclude incidental occurrence. Representative pictures are shown in the publication. Statistical analysis for automatized cell size analysis was performed using the GraphPad Prism software, version 6.01 (GraphPad Prism, Inc., La Jolla, USA) for parametric comparison of two groups. An unpaired *t* test was applied to compare the mean cell size of attached cells on the processed samples. Results were considered statistically significant when *p* < 0.05 (*p < 0.05, ***p < 0.001, and ****p < 0.0001).

## Results

### Cryofixation and sectioning of un-/cellularized PCL/PLA scaffolds produced sections with moderate deformations

**Fig. 2:**
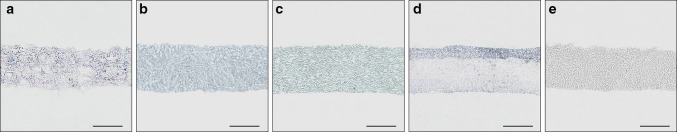
Comparison of different fixation and embedding methods of uncellularized PCL/PLA scaffolds. Bright field images of unstained sections. **a** Embedding and cryosectioning in OCT compound. **b** Stabilization with 10% gelatin, fixation in 3.7% PFA and standard paraffin embedding. **c** Stabilization with 25% gelatin, fixation in 3.7% PFA and standard paraffin embedding. **d** Fixation in 3.7% PFA and embedding in acrylic resin. **e** Fixation in 3.7% PFA and embedding in low-melting-point paraffin maintained optimal structure of uncellularized PCL/PLA membranes out of all variants of fixation and embedding. *PCL/PLA* polycaprolactone/polylactide, *OCT* optimal cutting temperature compound, *PFA* paraformaldehyde. Scale bars represent 100 µm

**Fig. 3:**
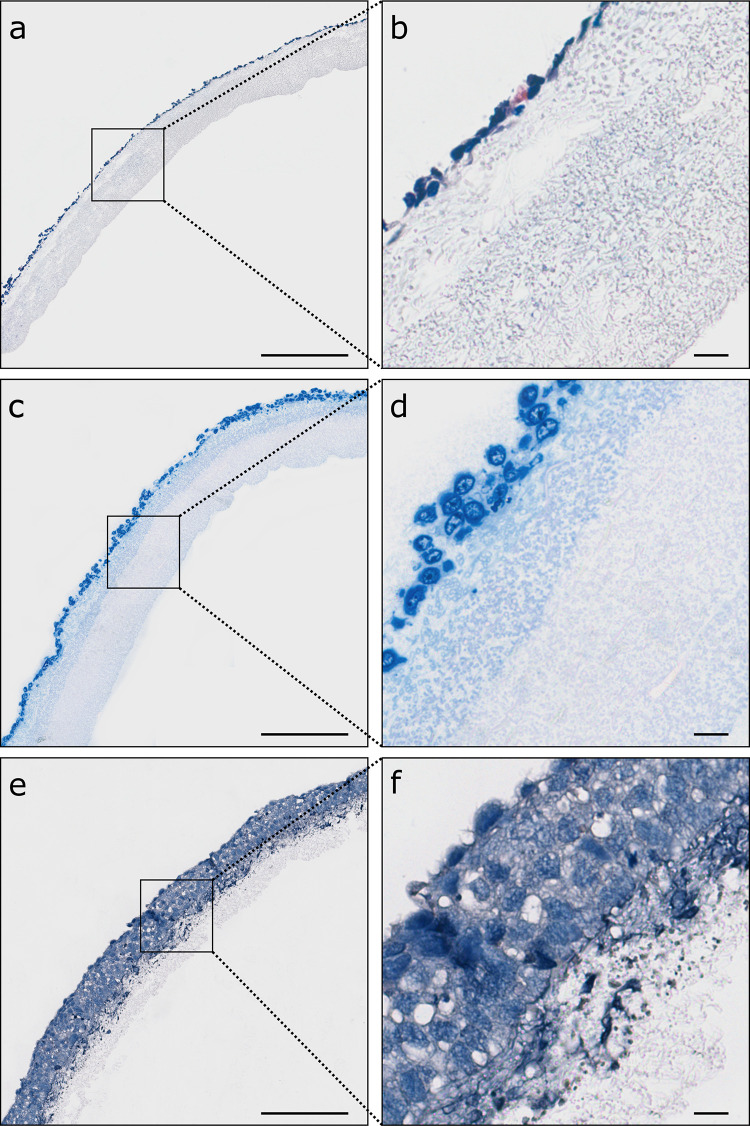
Comparison of different fixation and embedding methods of cellularized PCL/PLA membranes. Rows represent the respective embedding methods. Sections were stained and imaged with bright field imaging. **a, b** Embedding and cryosectioning in OCT compound; HE staining. **c, d** Fixation in 3.7% PFA, embedding in acrylic resin; toluidine blue staining. **e, f** Fixation in 3.7% PFA, embedding in low-melting-point paraffin (max. 50 °C); HE staining. *PCL/PLA* polycaprolactone/polylactide, *OCT* optimal cutting temperature compound, *PFA* paraformaldehyde, *HE* hematoxylin and eosin. Scale bars in **a, c, e** represent 200 µm, and those in **b, d, f** represent 20 µm

Cryofixation and sectioning of uncellularized PCL/PLA scaffolds was possible and created sections with spots of shrunken and diminished fibers throughout the specimen. No differences in fiber structures were seen between cryosections of uncellularized and cellularized PCL/PLA scaffolds (Figs. 2a, 3a, b). However, attached cells appeared reduced in their size compared to the cells attached on PCL/PLA scaffolds processed by low-melting-point paraffin embedding (Fig. 7 b, d, e).

### Acrylic resin embedding of un-/cellularized PCL/PLA scaffolds is limited to 3 µm sections

Acrylic resin processed un-/cellularized membranes displayed fiber structures that were unchanged in size and diameter (Figs. 2d, 3c, d). Coarse- and fine-meshed structures can be clearly distinguished. However, it was not possible to produce sections with a thickness of more than 3 µm.

### Automatized dehydration and standard paraffin embedding led to melted PCL/PLA scaffolds

Automatized dehydration and paraffin embedding of fixed un-/cellularized PCL/PLA membranes resulted in a dissolved PCL/PLA mesh because of temperatures up to 61 °C. An increase in time for PCL/PLA scaffold fixation from 1 to 24 h strengthened the material, but did not influence the thermostability of PCL/PLA.

### Combination of gelatin (10% and 25%) stabilization and standard paraffin embedding increased thermostability of uncellularized but not of cellularized PCL/PLA scaffolds

Gelatin stabilization of uncellularized PCL/PLA membranes with concentrations of 10% and 25% gelatin before embedding in standard paraffin (up to 61 °C) increased the stability of PCL/PLA scaffolds to a certain extent, whereas gelatin concentrations of 5% did not enhance thermostability and resulted in a dissolved PCL/PLA mesh. Sections of membranes treated with a gelatin concentration of 10% and 25% in combination with standard paraffin embedding showed partly shrunken and melted fibers, but an overall improved appearance compared to uncellularized and unprocessed PCL/PLA scaffolds (Fig. 2b, c). However, microtome sectioning of cellularized PCL/PLA membranes stabilized with 5%, 10% or 25% gelatin did not produce any utilizable paraffin sections. Stabilization of cellularized PCL/PLA membranes with a gelatin concentration of 5% resulted in a dissolution of PCL/PLA fibers. Membrane stabilization with concentrations of 10% and 25% gelatin enhanced the detachment of the gelatin core from the surrounded paraffin block.

### Low-melting-point paraffin embedding of un-/cellularized PCL/PLA scaffolds produced sections with PCL/PLA fibers unchanged in size or diameter

Embedding of un-/cellularized PCL/PLA scaffolds with low-melting-point paraffin (max. 50 °C) by microwave tissue processing produced comparable sections of PCL/PLA scaffolds. Scaffold and fibers appeared stable and solid as fiber diameter and pore size remained unchanged (Fig. 2e). In addition, cellularized membranes revealed characteristic cell morphology (Figs. 3e, f, 7 a, c, e). The shape and size of the cells appeared similar to cells cultivated in conventional cell culture. Therefore, this technique was used as the method of choice for further procedures.

### Enzymatic antigen retrieval of low-melting-point paraffin-embedded cellularized PCL/PLA scaffolds with pepsin led to intense and specific immunohistochemically staining of CK7

**Fig. 4:**
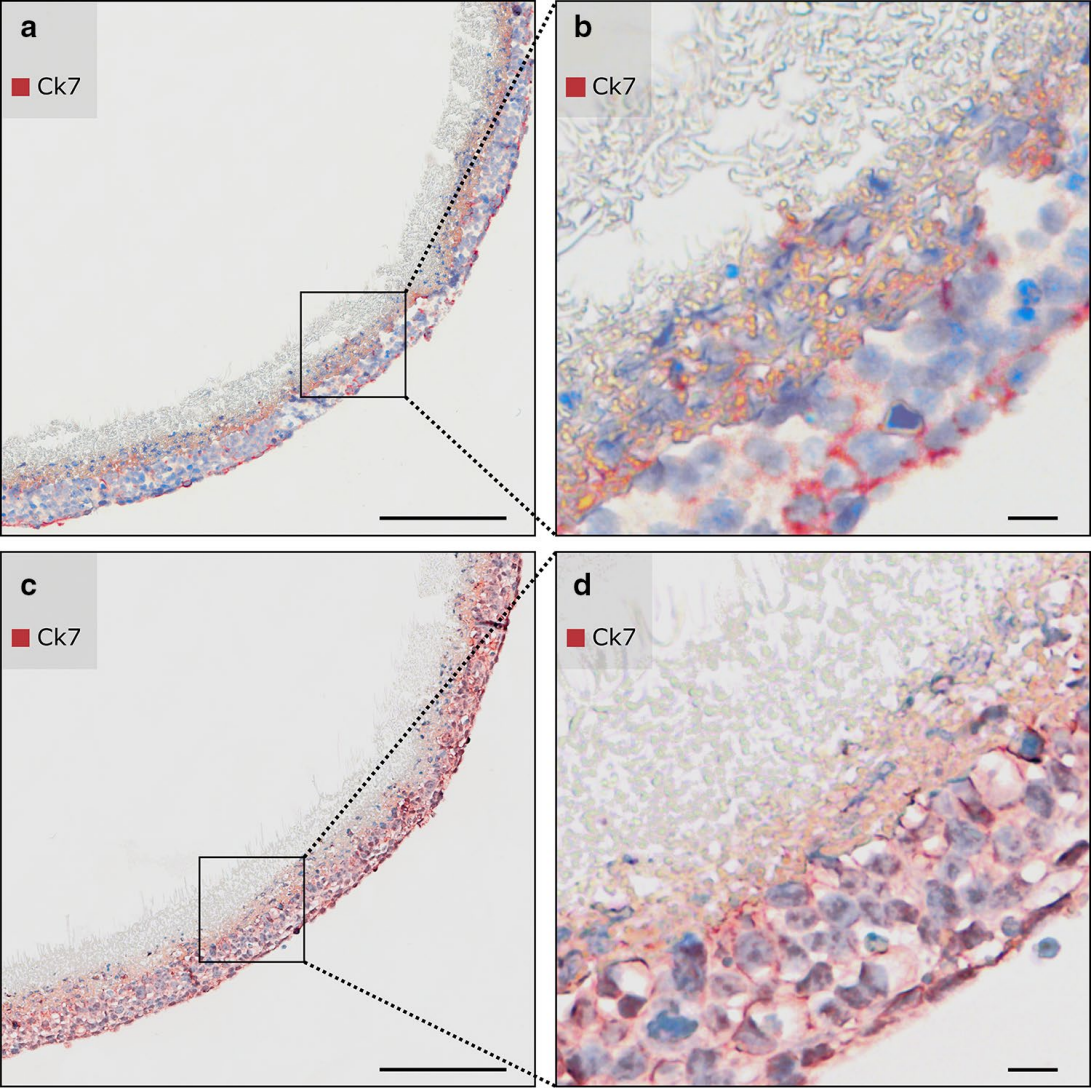
Enzymatic antigen retrieval of PCL/PLA membranes cellularized with trophoblast cells. Columns represent different enzymatic antigen retrievals. Membranes were fixed in 3.7% PFA and embedded in low-melting-point paraffin (max. 50 °C). Sections were immunostained with anti-CK7. Nuclei were counterstained with hematoxylin. Sections were imaged with bright field. **a, b** Antigen retrieval with proteinase K digestion for 30 min reveals unspecific staining of CK7 with poor cellular integrity. **c, d** Antigen retrieval with pepsin digestion for 30 min provides specific immunohistochemically staining of CK7 and optimal morphology of cellularized PCL/PLA membranes. *CK7* Cytokeratin 7, *PCL/PLA* polycaprolactone/polylactide, *PFA* paraformaldehyde. Scale bars in **a, c** represent 200 µm and in **b, d** 20 µm

Trophoblast cellularized membranes fixed in 3.7% PFA, embedded in low-melting-point paraffin, and pretreated with proteinase K for antigen retrieval revealed irregular an insufficient staining of CK7 with poor cellular integrity (Fig. 4a, b). In contrast, the most intense and specific immunohistochemically staining of CK7 was observed using an enzymatic pretreatment with pepsin (Fig. 4c, d). Pepsin digestion appeared to be the most effective antigen retrieval method to treat cellularized, temperature-sensitive PCL/PLA membranes. Hence, this technique proved to be the method of choice for further experiments requiring antigen retrieval. Rabbit immunoglobulins served as a negative control.

### Immunofluorescence revealed migration of trophoblast cells in low-melting-point paraffin-embedded PCL/PLA scaffolds and successful staining of CK7

**Fig. 5:**
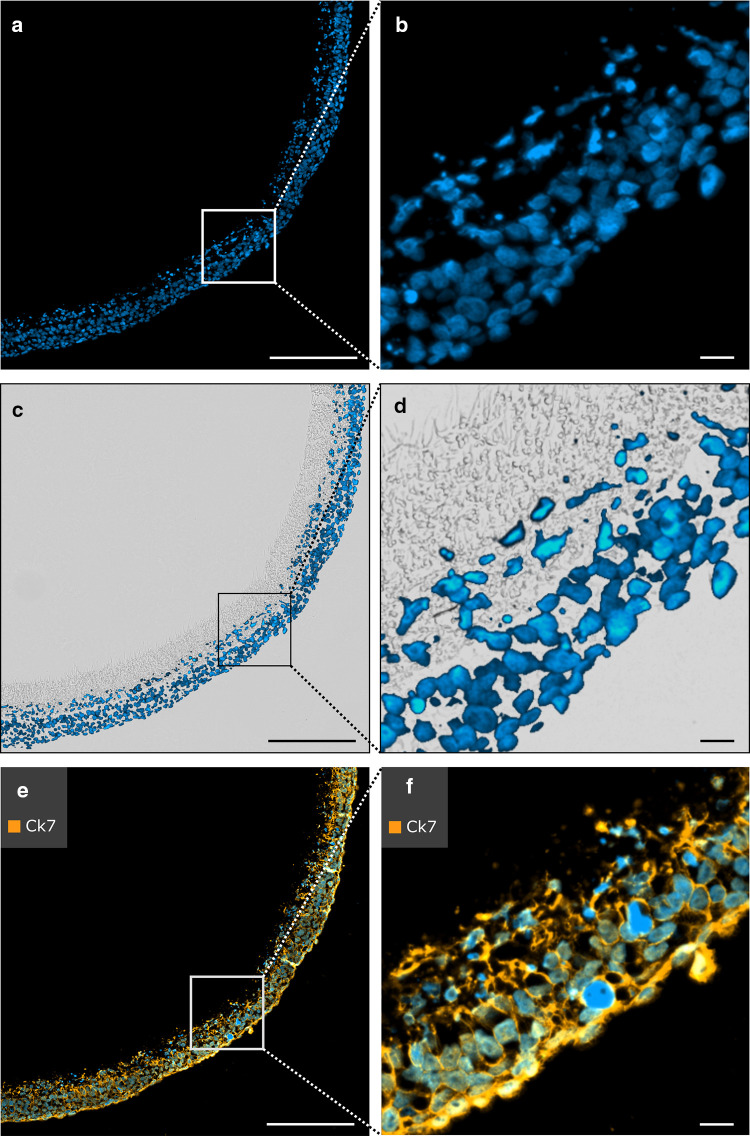
Immunofluorescent staining of cellularized PCL/PLA membranes cellularized with trophoblast cells. Sections were fixed in 3.7% PFA, embedded in low-melting-point paraffin (max. 50 °C), and antigen retrieval was performed with pepsin for 30 min. Nuclei were counterstained with DAPI (blue). **a, b** DAPI staining reveals successful cellularization of the PCL/PLA membrane. **c, d** Overlay image with fluorescence and bright field microscopy. Trophoblast cell migration into the PCL/PLA membrane is visualized with DAPI staining. **e, f** Sections are immunostained with anti-CK7. CK7 is used with Alexa Fluor 633 secondary antibody. Immunofluorescence demonstrates positive expression of CK7 in trophoblast cells. *CK7* cytokeratin 7, *PCL/PLA* polycaprolactone/polylactide, *PFA* paraformaldehyde. Scale bars in **a, c, e** represent 200 µm, and those in **b, d, f** represent 20 µm

DAPI staining confirmed successful cellularization of PCL/PLA membranes with ACH-3P cells (Fig. 5a, b). ACH-3P cells migrated to the center of the PCL/PLA membrane (Fig. 5c, d). Cells stopped migration at the coarse-meshed/fine-meshed layer interface, since the cell size is too large for invading the fine-meshed fiber network. In addition, immunofluorescence of CK7 showed distinct staining of ACH-3P cells (Fig. 5e, f).

### In situ padlock probe detection in combination with immunostaining for CK7 was feasible in low-melting-point paraffin-embedded PCL/PLA scaffolds

**Fig. 6:**
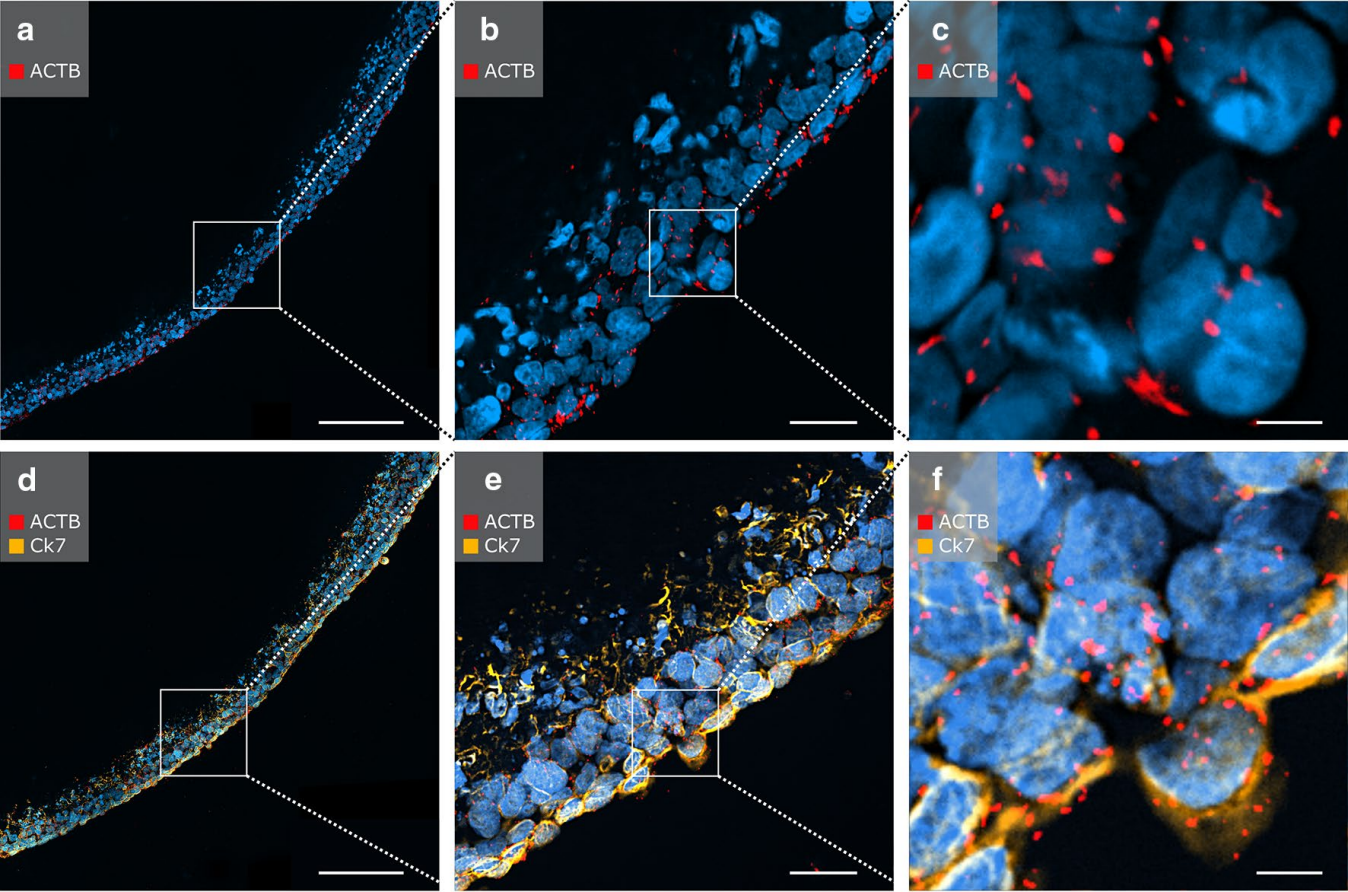
In situ padlock probe method applied on cellularized PCL/PLA membranes. Nuclei were counterstained with DAPI (blue). **a**–**c** In situ detection of ACTB mRNA transcripts using padlock probes. Single dots represent ACTB transcripts. **d**–**f** In situ detection of ACTB (red) mRNA transcripts using padlock probes combined with immunofluorescent staining of CK7 (orange). Scale bars in **a, d** represent 200 µm, those in **b, e** represent 50 µm, and those in **c, f** represent 10 µm. *ACTB* beta actin, *CK7* cytokeratin 7, *PCL/PLA* polycaprolactone/polylactide, *DAPI* 4′,6-diamidino-2-phenylindole

After pepsin digestion, the cellularized PCL/PLA scaffolds were processed for in situ padlock probe technology and immunofluorescent staining of CK7. Single treatment with in situ padlock probe technology for ACTB mRNA transcripts displayed clear ACTB signals appearing as red fluorescent dots (Fig. 6a–c). The combination of in situ padlock probe technology and immunofluorescence offers ideal conditions for effective spatial discrimination between mRNA transcripts and proteins. The cells showed expression of CK7 (orange fluorescent signals) and exhibited clear ACTB signals (red fluorescent signals) present at the border of the cell nucleus (Fig. 6d–f). Negative controls showed no staining for CK7 and no expression of ACTB (data not shown). Thus, in situ padlock probes can be successfully combined with immunostaining on cellularized thermosensitive PCL/PLA membranes.

### Low-melting-point paraffin-embedded PCL/PLA scaffolds preserved cell specific morphology

**Fig. 7:**
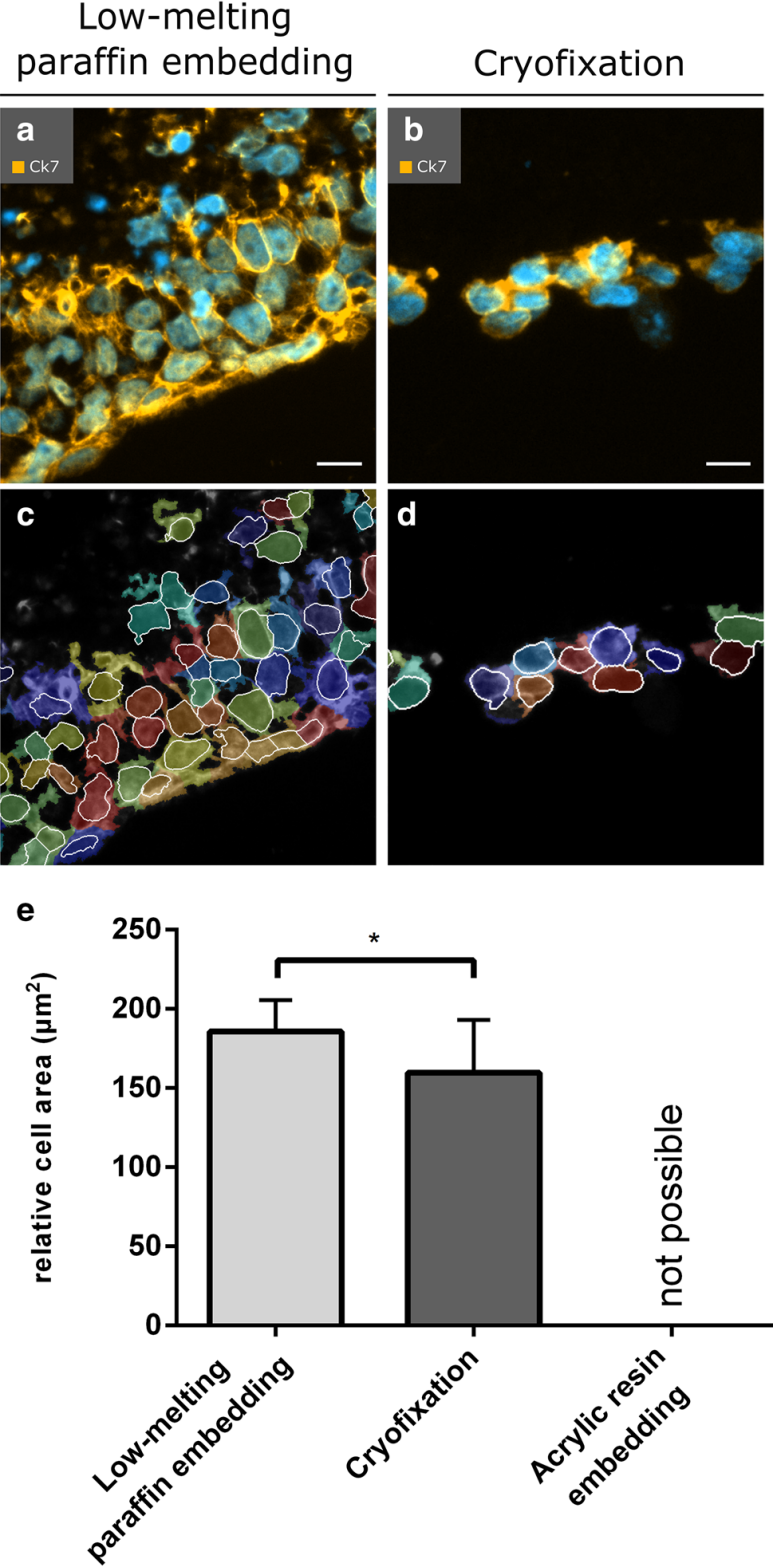
Automatized analysis of cells attached on PCL/PLA scaffolds after low-melting-point paraffin embedding and cyrofixation. Overlay images of the immunofluorescent channels DAPI (nuclei, blue) and Cy5 (intermediate filaments, orange) of **a** low-melting-point paraffin-embedded and **b** cryofixed samples are shown. **c, d** Output images of the quantification of cell nuclei and cell area by the CellProfiler software are displayed. The colored areas show the cellular regions. Cell nuclei are indicated by white frames. **e** The relative cell area of each embedding method shows the difference between the embedding methods. (*p < 0.05). *CK7* cytokeratin 7; Scale bars in **a, b** represent 20 µm

We did an automatized cell analysis to validate the influence of selected embedding methods on the cell specific morphology. Analysis of the cell size revealed that low-melting paraffin embedding preserved cell morphology much better than cyrofixation. Thus, after cyrofixation the average cell size was reduced by 14% compared to the cell size after low-melting paraffin embedding, 160 ± 8 µm^2^ and 186 ± 6 µm^2^ (*p* = 0.0351), respectively (Fig. 7).

## Discussion

The treatment of thermosensitive materials requires adaption of analyzing methods such as protocols for histological embedding and sectioning. Due to the properties of PCL/PLA scaffolds, processing temperatures must be kept below the material’s melting temperature of 51 °C to retain scaffold structure and cell–membrane interactions. However, in addition to cryoembedding, most of the histological embedding methods include working steps at temperatures above 61 °C. In addition, antigen retrieval is often necessary for epitope demasking for staining techniques such as immunohistochemistry or immunofluorescence on the tissue to retain specific antibody staining. Standard antigen retrieval is performed in a decloaking chamber™ (Biocare Medical), which works at 120 °C.

There is hardly any literature available that offers solutions for avoiding the use of embedding methods that require high heat exposure to thermosensitive samples. Hence, most of the studies completely lack histological analysis due to material properties.

**Fig. 8:**
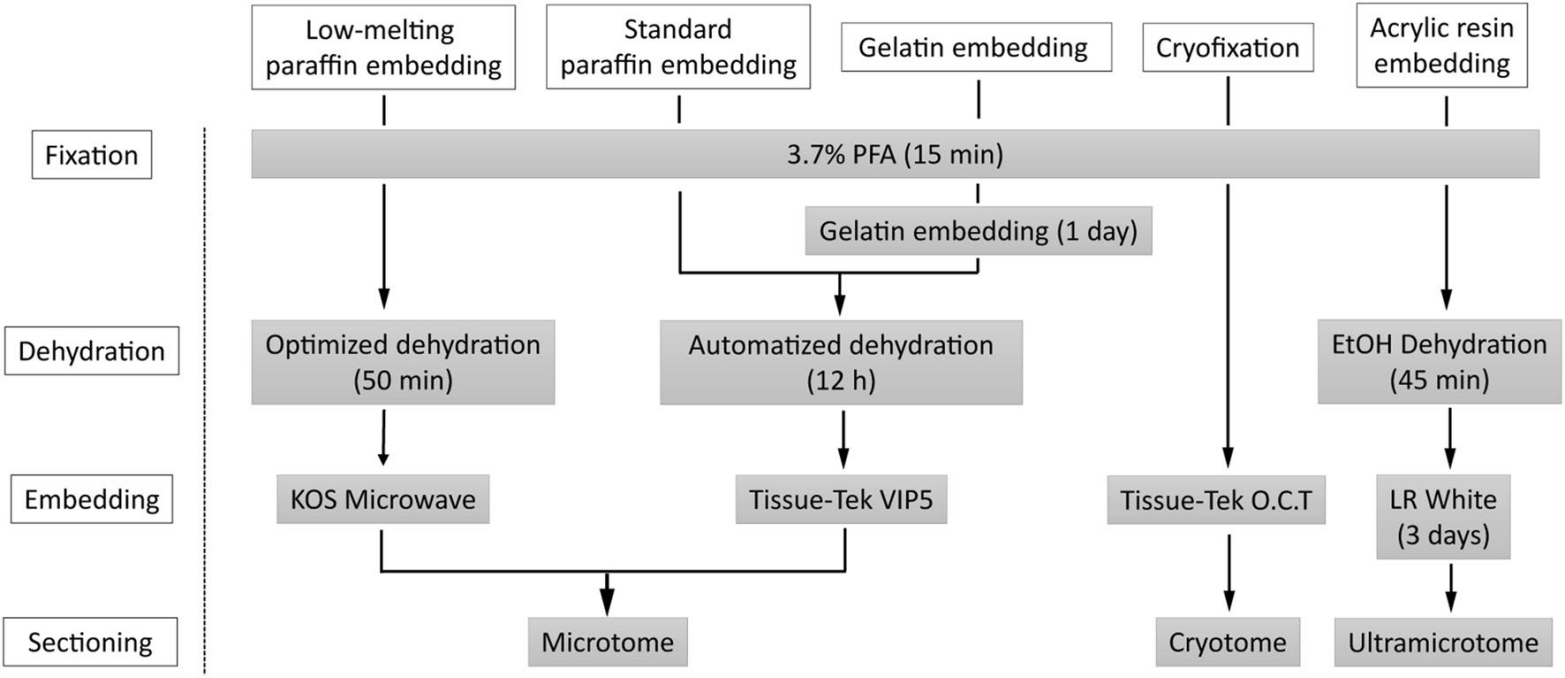
Scheme of the workflow of histological techniques for thermosensitive materials

We compared several histological techniques for thermosensitive materials, such as cryofixation, gelatin embedding followed by automatized dehydration and standard paraffin embedding, and an acrylic resin embedding method, and the established method of low-melting-point paraffin embedding of electrospun PCL/PLA scaffolds (Fig. 8).

Cryofixation seems to be the method of choice for such applications, but compared to paraffin-embedded sections, frozen tissue sections are usually thicker, which results in low microscopic resolution and a reduced ability to capture detailed tissue morphology. Nevertheless, cryofixation is thought to better preserve antigen and antigenicity.

According to the method of Hruschka et al. we embedded the PCL/PLA scaffold in different gelatin concentrations before embedding in standard paraffin to improve heat stability (Hruschka et al. 2013). The simplicity of the gelatin-embedding technique makes it attractive for numerous investigations in cell–material interactions. However, gelatin/paraffin sectioning introduced a major problem, as the unequal stiffness of the different materials resulted in the gelatin core separating from the paraffin.

These findings were also observed by the groups of Brown et al. and James et al. In comparison with mature natural tissues, cell-seeded scaffolds exhibit enormous spatial discontinuity during stages of cell proliferation. This discontinuity within cell–material interaction may be an explanation for problems during sectioning (Brown et al. 2005; Fox et al. 1985).

Acrylic resin embedding is generally performed at temperatures of 60 °C or higher. However, embedding at room temperature is possible, as the medium is used for immunolabeling as well (He et al. 2018; McDonald 2014). As this method is generally used for analysis of ultrastructures, cutting resulted in very thin sections (3 µm) of acrylic resin-embedded samples. Moreover, the high density of the resin does not allow standard histological staining, such as HE staining or immunohistochemistry, with the exception of toluidine blue staining, as the dye is able to penetrate resins (Lowe and Anderson 2015). In addition, the extensive processing timeline, up to several days, must also be considered.

Embedding of uncellularized and cellularized PCL/PLA scaffolds in low-melting-point paraffin at temperature of 50 °C resulted in fibers unchanged in diameter and structure and led to preserved scaffold formation. This method seemed to be the best available technique when compared with all the alternative variant fixation and embedding techniques, since it preserved an unchanged structure of unprocessed and cellularized PCL/PLA scaffolds.

Antigen retrieval reveals epitopes and other cellular targets for antibodies and oligonucleotides for immunohistochemistry or immunofluorescence after formaldehyde fixation (Robinson and Vandre 2001). Fixation with formaldehyde involves cross-linking of reactive sites within proteins and between different proteins via methylene bridges (Fox et al. 1985; Mason and O’Leary 1991). During these processes the tertiary and quaternary structures of proteins are modified, while primary and secondary structures are preserved (Mason and O’Leary 1991). The temperature and duration of fixation significantly reduces immunoreactivity for many antigens (Battifora and Kopinski 1986; Leong and Gilham 1989). In addition to heat-induced antigen retrieval, the enzymatic digestion with proteolytic enzymes, such as proteinase K and pepsin, is a useful alternative for avoiding heat-induced changes in the thermosensitive PCL/PLA scaffold (Ramos-Vara and Beissenherz 2000). The acting mechanism of antigen retrieval by enzymes has not been fully clarified. However, it is supposed that bonds formed between proteins and the fixative are nonspecifically cleaved (McNicol and Richmond 1998; Mighell et al. 1998). The duration and temperature of the digestion are determining factors for this effect (Battifora and Kopinski 1986). To evaluate which type of enzymatic antigen retrieval allows subsequent immunohistochemical and immunofluorescence staining the sections were stained for human CK7, a protein that is expressed in epithelial cells of various organs like ovary, lung, and breast tissues (Judson et al. 2008; Luo et al. 2017; Sati et al. 2010). Low-melting-point paraffin embedding and pepsin antigen retrieval appeared to be the most favorable techniques for visualization of cells and fiber structure of cellularized PCL/PLA scaffolds structures when compared among all of the variants relating to procedures of fixation, embedding and low-temperature antigen retrieval methods. This method allows histological analysis of the attached cells and assessment of cell–scaffold interactions. In addition, it was possible to apply the in situ padlock probe method, a specific mRNA-based staining technique, to the low-melting-point paraffin-embedded and enzymatically treated samples (El-Heliebi et al. 2017; Larsson et al. 2010). We performed the in situ padlock probe approach on our samples by targeting the mRNA transcripts for the housekeeping ACTB. Combining this method with immunofluorescence for CK7 resulted in positive signals from both techniques. All tested methods, including immunohistochemistry, immunofluorescence, and the in situ padlock probe technique, revealed reproducible results.

Low-melting-point paraffin embedding with enzymatic antigen retrieval represents an inexpensive and convenient technique that is perfectly suited to histological processes involving thermosensitive material with complex physical properties. In conclusion, this generally applicable method offers an alternative to standard preparations for histological determinations in a variety of sensitive samples.
